# Supplementary material for: Plakoglobin is a mechanoresponsive regulator of naive pluripotency
Source: Nat Commun. 2023 Jul 7;14:4022. doi: 10.1038/s41467-023-39515-0 (PMC10329048; doi:10.1038/s41467-023-39515-0)
Supplement: Supplementary file 1 — Supplementary Information [file 41467_2023_39515_MOESM1_ESM.pdf]

Supplementary Information

Plakoglobin is a mechanoresponsive regulator of naïve pluripotency

Timo N. Kohler[1,2,#], Joachim De Jonghe[1,#], Anna L. Ellermann[1,#], Ayaka Yanagida[2,5,6], Michael Herger[1], Erin M. Slatery[3,4], Antonia Weberling[1,3], Clara Munger[1,3,4], Katrin Fischer[1], Carla Mulas[2,7,11], Alex Winkel[3], Connor Ross[2,3,8], Sophie Bergmann[3,4], Kristian Franze[3,9,10], Kevin Chalut[2,11], Jennifer Nichols[2,3,8], Thorsten E. Boroviak[2,3,4]*, Florian Hollfelder[1]*

[1]Department of Biochemistry, University of Cambridge, Hopkins Building, Tennis Court Road, Cambridge CB2 1QW, United Kingdom

[2]Wellcome Trust – Medical Research Council Stem Cell Institute, University of Cambridge, Jeffrey Cheah Biomedical Centre, Puddicombe Way, Cambridge CB2 0AW, United Kingdom

[3]Department of Physiology, Development and Neuroscience, University of Cambridge, Cambridge CB2 3DY, United Kingdom

[4]Centre for Trophoblast Research, University of Cambridge, Cambridge CB2 3EG, United Kingdom

[5]Department of Veterinary Anatomy, Graduate School of Agriculture and Life Sciences, The University of Tokyo, Tokyo 113-8657, Japan

[6]Stem Cell Therapy Laboratory, Advanced Research Institute, Tokyo Medical and Dental University, 1-5-45 Yushima, Bunkyo-ku, Tokyo 113-8510, Japan

[7]Randall Centre for Cell and Molecular Biophysics, King's College London, London, SE1 1UL, UK

[8]MRC Human Genetics Unit, Institute of Genetics and Cancer, The University of Edinburgh, Crewe Road, Edinburgh, EH4 2XU, UK

[9]Institute of Medical Physics, Friedrich-Alexander-Universität Erlangen-Nürnberg, Henkestr. 91, 91052 Erlangen, Germany.

[10]Max-Planck-Zentrum für Physik und Medizin, 91054 Erlangen, Germany

[11]Present address: Altos Labs, Cambridge Institute of Science, Cambridge, UK

* Corresponding authors: fh111@cam.ac.uk, teb45@cam.ac.uk

# Authors contributed equally

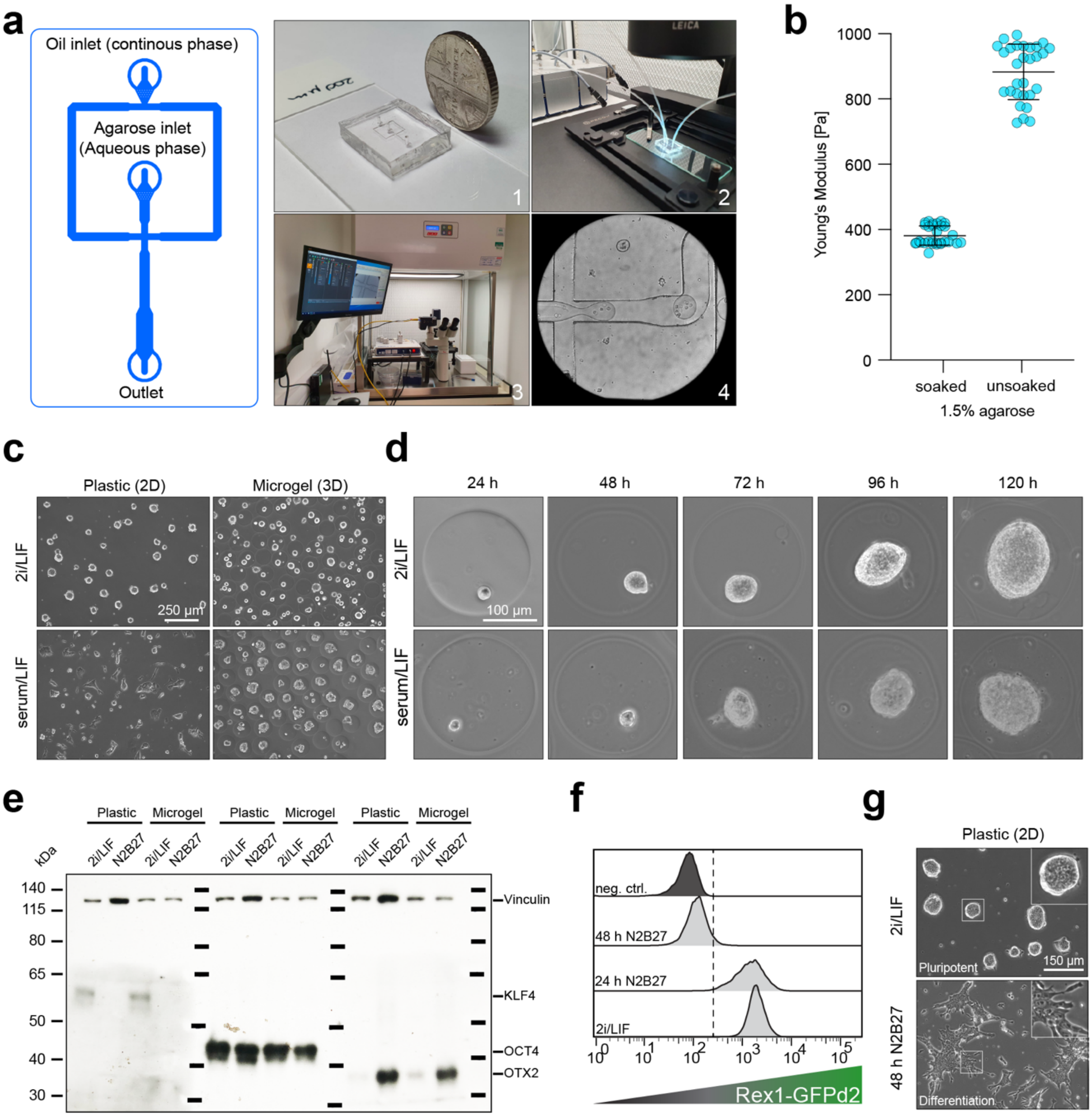

a
Oil inlet (continous phase)
Agarose inlet
(Aqueous phase)
Outlet
b
Young's Modulus [Pa]
1000
800
600
400
200
0
soaked
unsoaked
1.5% agarose
c
Plastic (2D)
Microgel (3D)
2i/LIF
serum/LIF
250 µm
d
24 h
48 h
72 h
96 h
120 h
2i/LIF
serum/LIF
100 µm
e
Plastic
Microgel
2i/LIF
N2B27
kDa
140
115
80
65
50
40
30
Vinculin
KLF4
OCT4
OTX2
f
neg. ctrl.
48 h N2B27
24 h N2B27
2i/LIF
Rex1-GFPd2
g
Plastic (2D)
2i/LIF
48 h N2B27
Pluripotent
Differentiation
150 µm

Supplementary Fig. 1 | Microfluidic-based cell encapsulation. a, Schematic of the microfluidic flow-focusing device (FFD) and explanatory images of the encapsulation rig set-up: (1) Single-inlet FFD, (2) Fully connected FFD with oil and agarose inlet and outlet, (3) Microfluidic encapsulation rig in laminar flow hood and (4) FFD crossing of aqueous (agarose) and continuous (oil) phase. **b**, Measurements of the elastic modulus of the ultra-low melting agarose by atomic force microscopy. Measurements were performed on bulk gels that were pre-incubated with medium (soaked) or without pre-incubation. Error bars indicate the mean and standard deviations; representative of 3 independent experiments**. c**, Low magnification phase contrast overview images of mESCs in 2i/LIF and serum/LIF cultured on tissue culture plastic or in microgels. Representative of 3 independent experiments **d**, Time course phase contrast images of encapsulated mESCs after 24 h, 48 h, 72 h, 96 h and 120 h of culture in naïve pluripotent (2i/LIF) and metastable pluripotent (serum/LIF) conditions. Representative of 3 independent experiments **e**, Western blot analysis of mESCs cultured in 2i/LIF (naïve pluripotency) or for 48 h in N2B27 (differentiation) on tissue culture plastic or within microgels. Analysis provided for the expression of KLF4 (naïve pluripotency marker), OCT4 (general pluripotency marker) and OTX2 (early differentiation marker). Vinculin was used as loading control. Representative of 3 independent experiments. **f**, Flow cytometric analysis of the RGd2 cells during pluripotency (2i/LIF) 24 h and 48 h of differentiation (N2B27). Cells have fully exited after 48 h of differentiation as indicated by the complete loss of Rex1-GFPd2. **g**, Phase contrast images of mESCs cultured on conventional tissue culture plastic in 2i/LIF (pluripotent) and 48 h of culture in N2B27 (differentiation). Representative of 3 independent experiments.

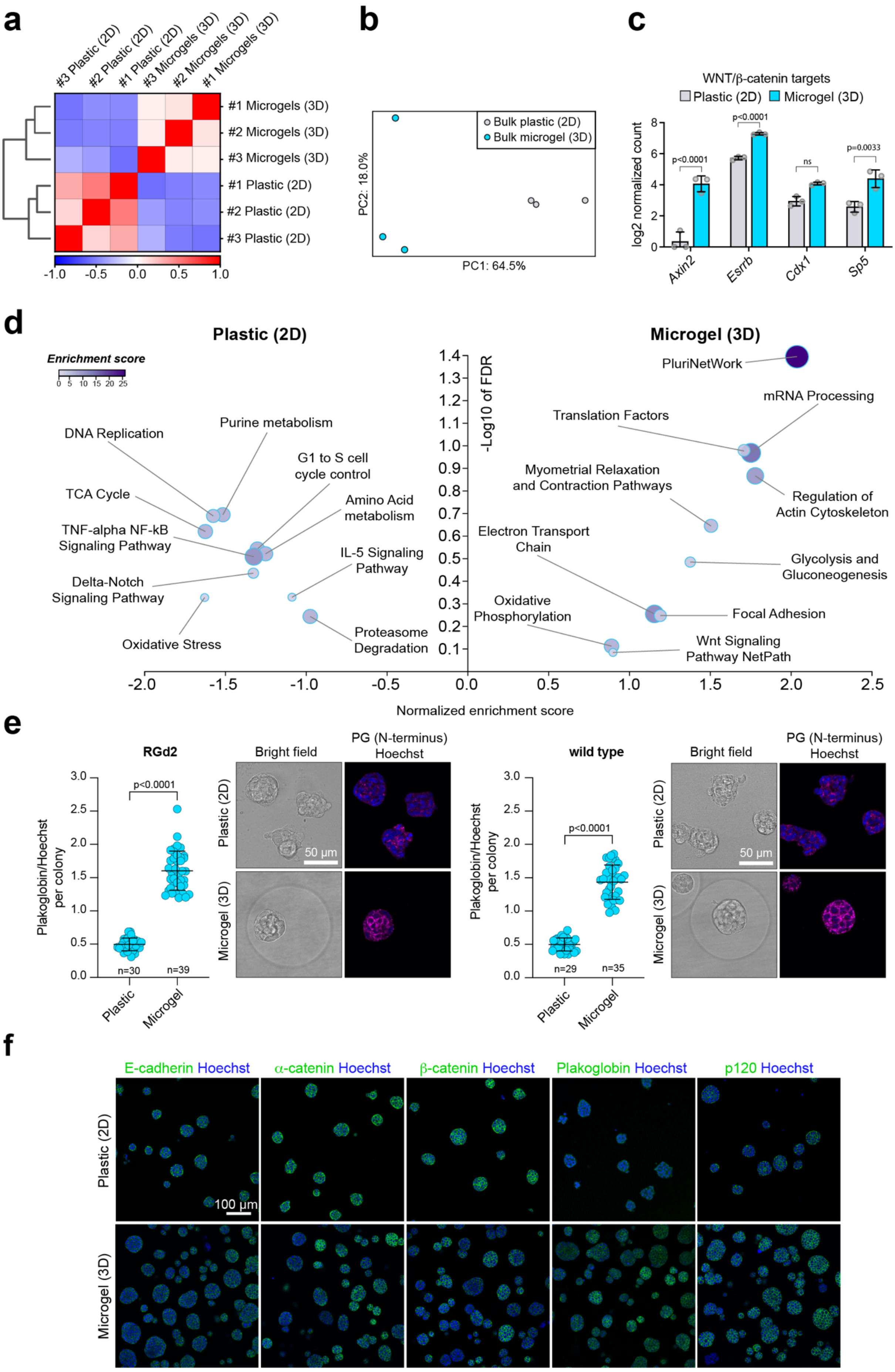
a
#3 Plastic (2D)
#2 Plastic (2D)
#1 Plastic (2D)
#3 Microgels (3D)
#2 Microgels (3D)
#1 Microgels (3D)
-1.0 -0.5 0.0 0.5 1.0
b
Bulk plastic (2D)
Bulk microgel (3D)
PC1: 64.5%
PC2: 18.0%
c
WNT/β-catenin targets
Plastic (2D) Microgel (3D)
log2 normalized count
p<0.0001
p<0.0001
ns
p=0.0033
Axin2
Esrrb
Cdx1
Sp5
d
Plastic (2D)
Microgel (3D)
Enrichment score
-Log10 of FDR
Normalized enrichment score
PluriNetWork
mRNA Processing
Translation Factors
Myometrial Relaxation and Contraction Pathways
Regulation of Actin Cytoskeleton
Electron Transport Chain
Glycolysis and Gluconeogenesis
Oxidative Phosphorylation
Focal Adhesion
Wnt Signaling Pathway NetPath
DNA Replication
Purine metabolism
G1 to S cell cycle control
TCA Cycle
Amino Acid metabolism
TNF-alpha NF-kB Signaling Pathway
IL-5 Signaling Pathway
Delta-Notch Signaling Pathway
Proteasome Degradation
Oxidative Stress
e
RGd2
Plakoglobin/Hoechst per colony
p<0.0001
n=30
n=39
Plastic
Microgel
Bright field
PG (N-terminus) Hoechst
Plastic (2D)
Microgel (3D)
50 µm
wild type
p<0.0001
n=29
n=35
f
E-cadherin Hoechst
α-catenin Hoechst
β-catenin Hoechst
Plakoglobin Hoechst
p120 Hoechst
Plastic (2D)
Microgel (3D)
100 µm

Supplementary Fig. 2 | Comparison of cell culture in microgels (3D) and on plastic (2D). **a**, Pearson correlation coefficient heatmaps for each bulk RNA-seq replicate sample for both mESCs seeded on plastic (2D, N=3) or encapsulated in microgels (3D N=3) under naïve pluripotent (2i/LIF) culture conditions. **b**, Principal component (PC; PC1=64.5%, PC2=18.0%) dimensional reduction plot of each sequenced replicate from mESCs cultured in 2i/LIF on plastic or encapsulated in microgels. **c**, Bulk RNA-seq analysis of known WNT/β-catenin target genes. Error bars indicate the mean and standard deviations and statistical significance levels across 3 replicates per condition are Bonferroni-adjusted *p*-values computed via differential gene expression analysis using the two-tailed DESeq2 tool. **d**, Gene set enrichment analysis using the most differentially expressed genes ($|\log_2$ fold-change$| > 0.5$ and Bonferroni-adjusted *p*-value < 10^$^{-3}$) between mESCs cultured on tissue culture plastic in 2i/LIF and in agarose microgels, highlighting a significant enrichment of the PluriNetWork (WikiPathways) under microgel culture conditions. The statistical test for differential expression analysis was a two-tailed DESeq2 across 3 replicates per condition. **e**, Confocal immunofluorescence images of naïve pluripotent (2i/LIF) mESCs (RGd2 (left) and wild type (right)) cultured on plastic and encapsulated in microgels stained for Plakoglobin (PG). Plakoglobin levels were quantified for colonies grown on plastic (RGd2 n=30 colonies; wild type n=29 colonies) and in microgels (RGd2 n=39 colonies; wild type n=35 colonies). Error bars indicate the mean and standard deviations. *p*-values were determined by a two-tailed unpaired *t*-test with Welch's correction. **f**, Confocal immunofluorescence images of naïve pluripotent (2i/LIF) cultured mESCs on plastic (2D) and encapsulated in microgels (3D). Cells were stained for E-cadherin, α-catenin, β-catenin, Plakoglobin, δ-catenin and nuclei with Hoechst. Scale bar: 100 µm. Representative of 3 independent experiments.

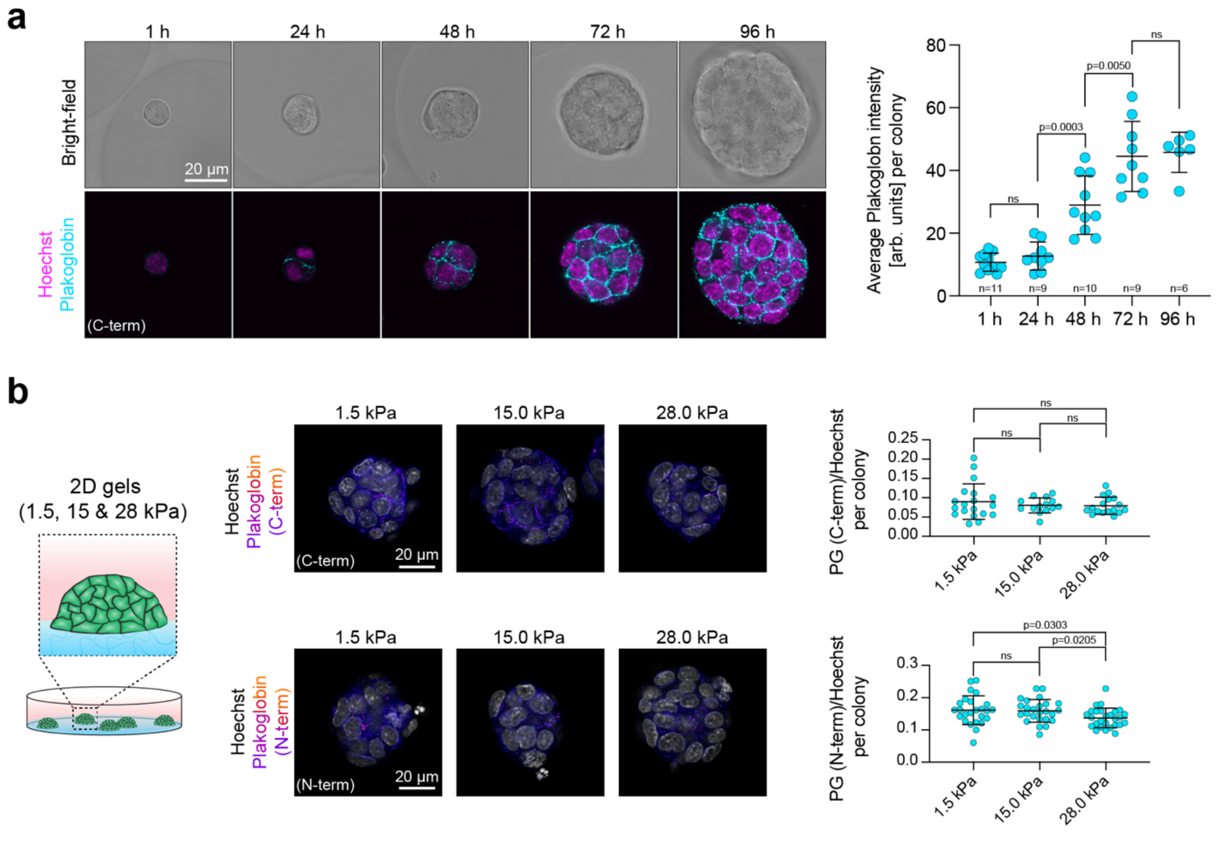

Supplementary Fig. 3 | Microenvironmental regulation of Plakoglobin. a, Representative confocal immunofluorescence images of a time-course over 96 h of naïve pluripotent (2i/LIF) microgel encapsulated mESCs. Cells were stained for Plakoglobin. Average Plakoglobin levels were quantified per colony, significant upregulation was observed as early as 48 h after encapsulation. Error bars indicate the mean and standard deviations. *p*-values were determined by a two-tailed unpaired *t*-test with Welch's correction. **b**, Confocal immunofluorescence images of naïve pluripotent (2i/LIF) mESCs cultured on commercially available (see methods) 2D polydimethylsiloxane gels with varying stiffnesses (1.5 kPa, 15 kPa and 28 kPa). Cells were stained for Plakoglobin (C-term and N-term). Plakoglobin levels (indicated on the right) remained mostly constant irrespective of 2D gel stiffness. Error bars indicate the mean and standard deviations. *p*-values were determined by a two-tailed unpaired *t*-test with Welch's correction; 3 independent experiments.

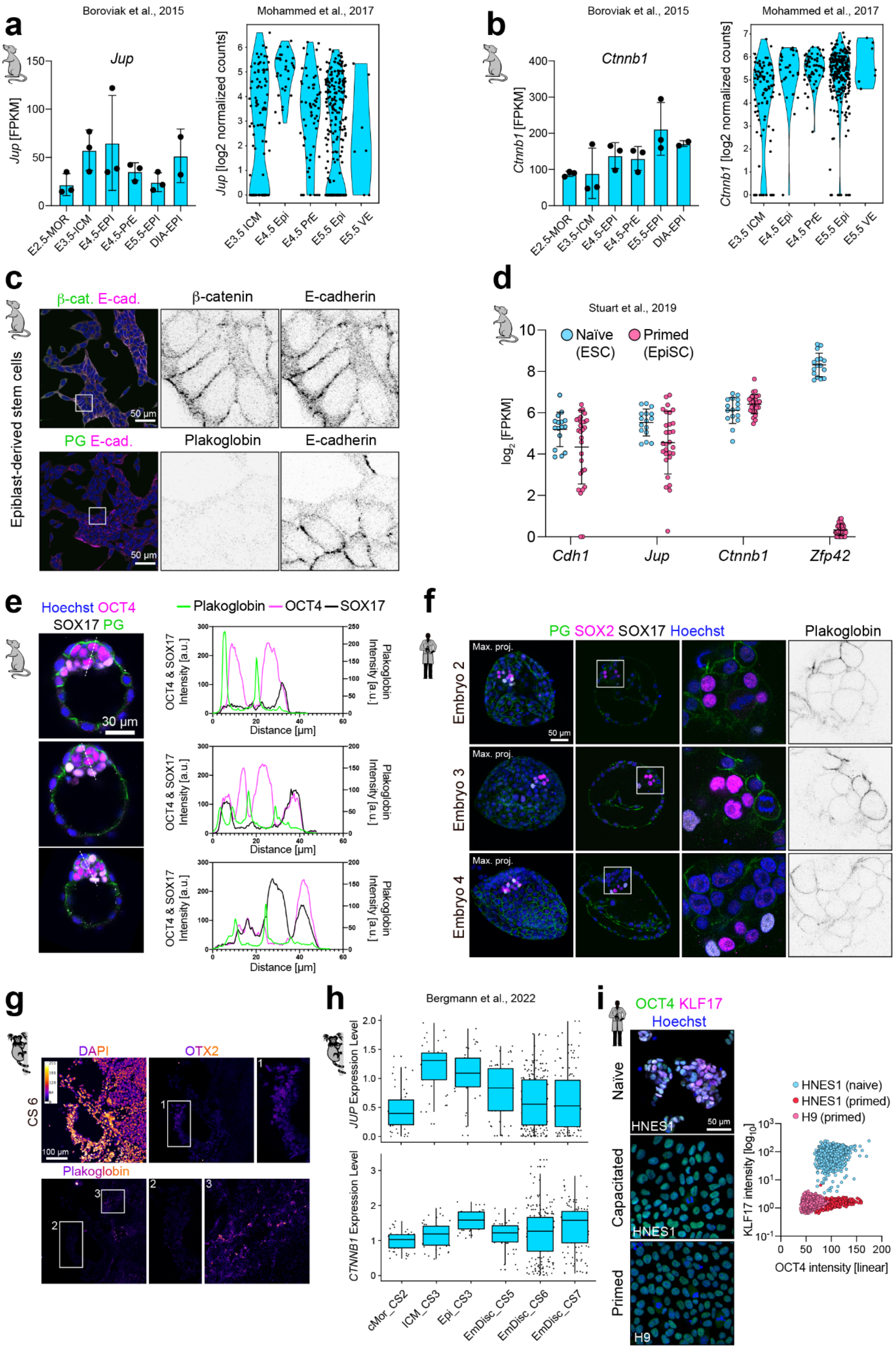
a
b
c
d
e
f
g
h
i
Boroviak et al., 2015
Mohammed et al., 2017
Jup
Jup [FPKM]
Jup [log2 normalized counts]
Ctnnb1
Ctnnb1 [FPKM]
Ctnnb1 [log2 normalized counts]
E2.5-MOR
E3.5-ICM
E4.5-EPI
E4.5-PrE
E5.5-EPI
DIA-EPI
E3.5 ICM
E4.5 Epi
E4.5 PrE
E5.5 Epi
E5.5 VE
Epiblast-derived stem cells
β-cat. E-cad.
β-catenin
E-cadherin
PG E-cad.
Plakoglobin
50 µm
Stuart et al., 2019
Naïve (ESC)
Primed (EpiSC)
log2 [FPKM]
Cdh1
Jup
Ctnnb1
Zfp42
Hoechst OCT4
SOX17 PG
30 µm
Plakoglobin
OCT4
SOX17
OCT4 & SOX17 Intensity [a.u.]
Plakoglobin Intensity [a.u.]
Distance [µm]
PG SOX2 SOX17 Hoechst
Max. proj.
Embryo 2
Embryo 3
Embryo 4
CS 6
DAPI
OTX2
100 µm
Bergmann et al., 2022
JUP Expression Level
CTNNB1 Expression Level
cMor_CS2
ICM_CS3
Epi_CS3
EmDisc_CS5
EmDisc_CS6
EmDisc_CS7
OCT4 KLF17
Hoechst
Naïve
Capacitated
Primed
HNES1
H9
HNES1 (naive)
HNES1 (primed)
H9 (primed)
KLF17 intensity [log10]
OCT4 intensity [linear]

Supplementary Fig. 4 | Plakoglobin expression in the embryo. a&b, Meta-analysis of RNA-seq data[10, 43] for *Jup* (Plakoglobin) and *Ctnnb1* (β-catenin) during early embryogenesis. MOR = morula, ICM = inner cell mass, EPI = Epiblast, DIA-EPI = diapause epiblast, PrE = primitive endoderm, VE = visceral endoderm. **c**, Confocal immunofluorescence images of mouse epiblast-derived stem cells (post-implantation) stained for β-catenin (β-cat.), Plakoglobin (PG) and E-cadherin. Scale bar: 50 µm. Representative of 3 independent experiments. **d**, Meta-analysis of single-cell RNA-seq data[48] of naïve mouse ESCs (pre-implantation) and primed post-implantation epiblast-derived stem cells (EpiSCs). **e**, Confocal immunofluorescence images of blastocysts (~E4.25) stained for Plakoglobin, OCT4 (epiblast marker) and SOX17 (primitive endoderm marker). Fluorophore intensities were quantified along the white dotted line marked in the merged image and are shown on the right. Primitive endoderm cells lost Plakoglobin expression once sorted to the inner cell mass' surface. Scale bar: 30 µm. **f**, Confocal immunofluorescence images of human blastocysts (Day 7) stained for SOX2 (epiblast marker), SOX17 (hypoblast marker) and Plakoglobin. Inserts show a magnified view of the inner cell mass. Scale bar: 50 µm. **g**, Immunofluorescence images of Carnegie stage 6 marmoset embryo stained for OTX2 and Plakoglobin. Inserts (1) and (2) show a magnified view of the embryonic disc and insert (3) of the maternal tissue. Scale bar: 100 µm. **h**, Meta-analysis of spatial RNA-Seq data[52] for *JUP* and *CTNNB1* expression in the marmoset embryo from Carnegie stage (CS) 2 to CS7. cMor = compacted morula, ICM = inner cell mass, Epi = epiblast, EmDisc = embryonic disc. In boxplots, the box represents interquartile range and median, and whiskers are drawn to minimum and maximum excluding outliers. **i**, Confocal immunofluorescence images of naïve, capacitated and primed hPSCs stained for OCT4 (general pluripotency marker) and KLF17 (primate-specific naïve pluripotency marker). Scale bar: 50 µm. Representative of 3 independent experiments.

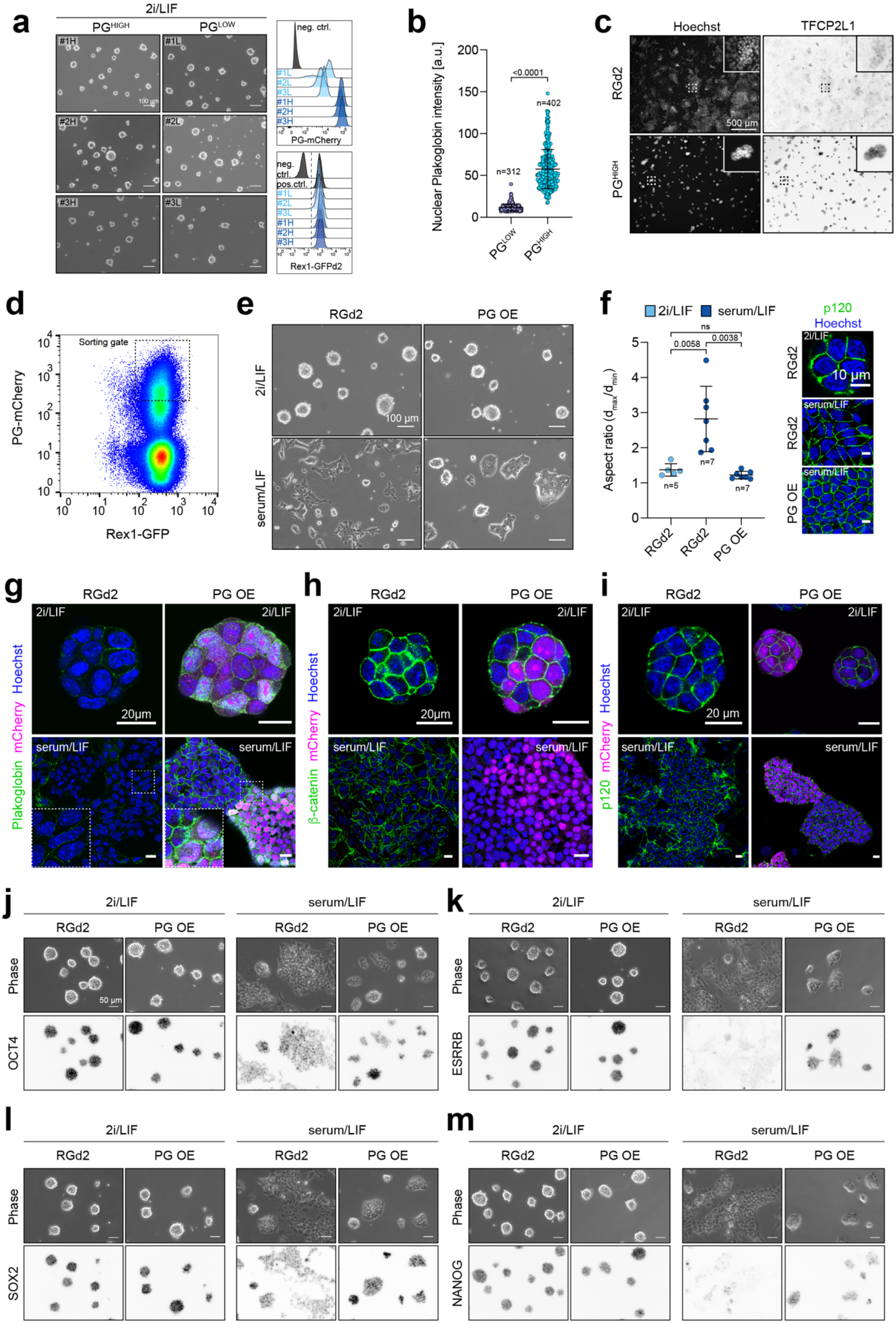

a
2i/LIF
PG^HIGH
PG^LOW
#1H
#1L
#2H
#2L
#3H
#3L
100 µm
neg. ctrl.
PG-mCherry
pos.ctrl.
Rex1-GFPd2
b
Nuclear Plakoglobin intensity [a.u.]
<0.0001
n=402
n=312
PG^LOW
PG^HIGH
c
Hoechst
TFCP2L1
RGd2
PG^HIGH
500 µm
d
PG-mCherry
Sorting gate
Rex1-GFP
e
RGd2
PG OE
2i/LIF
serum/LIF
100 µm
f
2i/LIF
serum/LIF
Aspect ratio (d_max/d_min)
ns
0.0058
0.0038
n=5
n=7
n=7
p120
Hoechst
10 µm
g
Plakoglobin mCherry Hoechst
20µm
h
β-catenin mCherry Hoechst
i
p120 mCherry Hoechst
20 µm
j
Phase
OCT4
50 µm
k
ESRRB
l
SOX2
m
NANOG

Supplementary Fig. 5 | Plakoglobin expression promotes naïve pluripotency in metastable culture conditions. a, Phase contrast images of clonally expanded PG^{LOW} (#1L, #2L, #3L) and PG^{HIGH} (#1H, #2H, #3H) cells cultured in 2i/LIF. Scale bar: 100 µm. **b**, Quantification of nuclear Plakoglobin immunofluorescence signal (ref. Fig.5D) in PG^{LOW} (n=312 cells) and PG^{HIGH} (n=402 cells) cells cultured under serum/LIF conditions. Error bars indicate the mean and standard deviations; *p*-values were determined by a two-tailed unpaired *t*-test with Welch's correction; 3 independent experiments. **c**, Epifluorescence images of RGd2 and PG^{HIGH} cells in metastable pluripotent conditions (serum/LIF) stained for the naïve pluripotency marker TFCP2L1. Scale bar: 500 µm. Representative of 3 independent experiments. **d**, Flow cytometric analysis of *CAG::Jup-2A-mCherry* transfected RGd2 cells. PG OE cells were sorted based on positive Rex1-GFPd2 and PG-mCherry signal (sorting gate indicated as a rectangle). **e**, Phase contrast images of RGd2 and PG OE cells cultured in naïve pluripotent (2i/LIF) and metastable pluripotent (serum/LIF) conditions. PG OE cells remained as tightly packed colonies under metastable conditions in serum/LIF similar to naïve cells in 2i/LIF. Scale bar: 100 µm. Representative of 3 independent experiments. **f**, Aspect ratio measurements ($distance_{max}/distance_{min}$) of RGd2 cells (in 2i/LIF n=5, in serum/LIF n=7) and PG OE cells (in serum/LIF n=7). Cell morphology is indicated by confocal immunofluorescence images on the right with cells stained for cortical p120. Error bars indicate the mean and standard deviations; *p*-values were determined by a two-tailed unpaired *t*-test with Welch's correction; 2 independent experiments. Scale bar: 10 µm. **g-i**, Confocal immunofluorescence images of RGd2 and PG OE cells in naïve pluripotent (2i/LIF) and metastable pluripotent (serum/LIF) conditions. Cells were stained for Plakoglobin (d), β-catenin (e) and p120 (f). Scale bar: 20 µm. Representative of 3 independent experiments. **j-m**, Epifluorescence and phase contrast images of RGd2 and PG OE cells under naïve pluripotent (2i/LIF) and metastable pluripotent (serum/LIF) conditions. Cells were stained for OCT4 (g), ESRRB (h), SOX2 (i) and NANOG (j). Scale bar: 50 µm. Representative of 3 independent experiments.

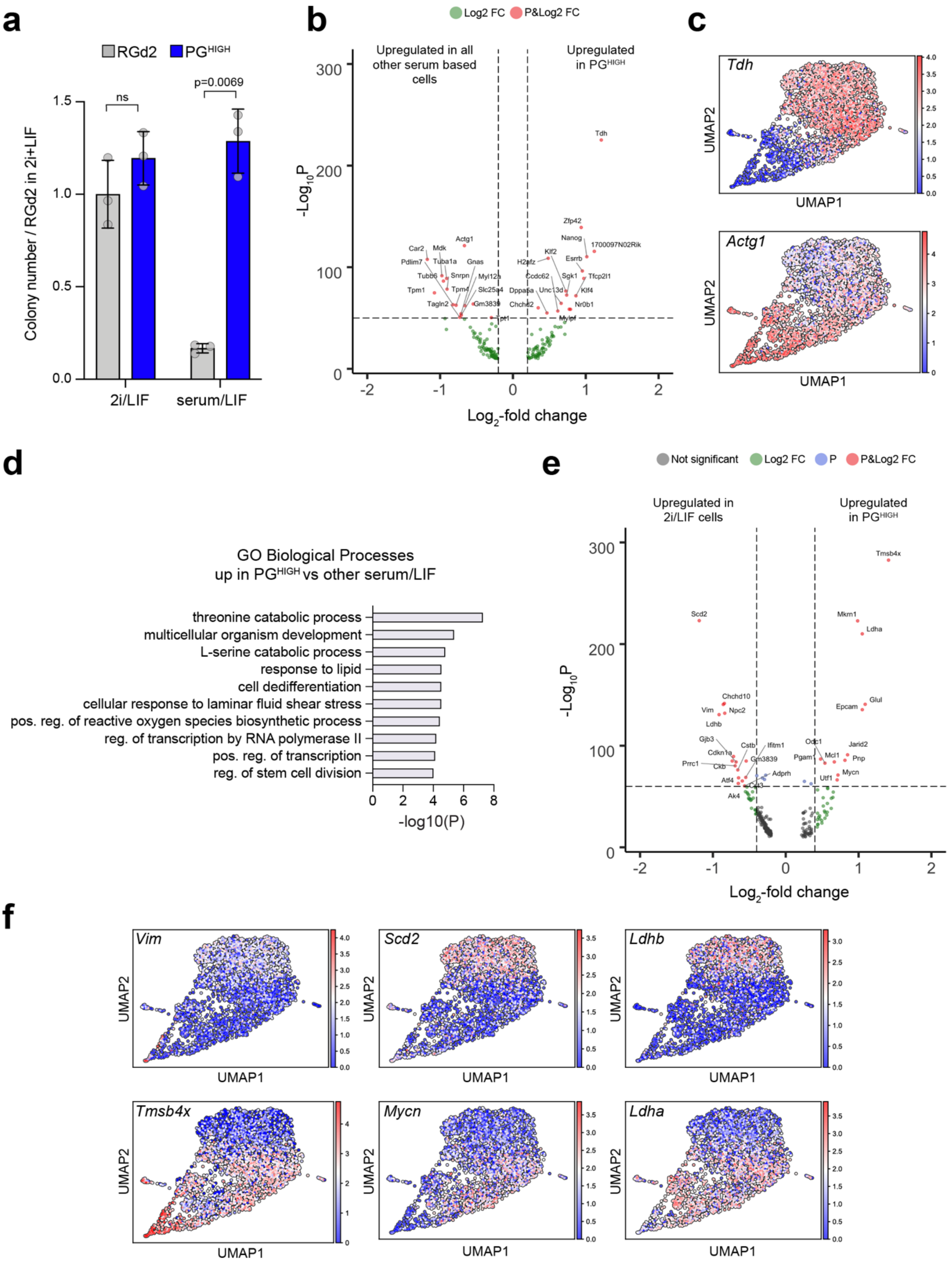
a
RGd2
PGHIGH
ns
p=0.0069
Colony number / RGd2 in 2i+LIF
2i/LIF
serum/LIF
b
Log2 FC
P&Log2 FC
Upregulated in all other serum based cells
Upregulated in PGHIGH
-Log10P
Log2-fold change
c
Tdh
Actg1
UMAP1
UMAP2
d
GO Biological Processes up in PGHIGH vs other serum/LIF
threonine catabolic process
multicellular organism development
L-serine catabolic process
response to lipid
cell dedifferentiation
cellular response to laminar fluid shear stress
pos. reg. of reactive oxygen species biosynthetic process
reg. of transcription by RNA polymerase II
pos. reg. of transcription
reg. of stem cell division
-log10(P)
e
Not significant
Log2 FC
P
P&Log2 FC
Upregulated in 2i/LIF cells
Upregulated in PGHIGH
-Log10P
Log2-fold change
f
Vim
Scd2
Ldhb
Tmsb4x
Mycn
Ldha
UMAP1
UMAP2

Supplementary Fig. 6 | Single-cell sequencing elucidates plakoglobin-induced re-establishment of naïve pluripotency. a, Colony formation assay of RGd2 and PG^{HIGH} cells. Error bars indicate the mean and standard deviations; (N=3)**. b**, Volcano plot of genes with $\log_2$-fold change > 0.2 and $-\log_{10}$(*p*-value) > 50, comparing PG^{HIGH} cells against all other serum/LIF cultured samples, calculated using a two-tailed Wilcoxon rank sum test. **c**, Gene-expression values projected on the UMAP plot for *Tdh* (upregulated in naïve 2i/LIF control cells and PG^{HIGH} in serum/LIF) and *Actg1* (upregulated in all other serum/LIF samples). **d**, List of Gene Ontology for Biological Processes upregulated in PG^{HIGH} cells compared to all other serum/LIF samples. **e**, Volcano plot of genes with $\log_2$-fold change > 0.4 and $-\log_{10}$(*p*-value) > 60, comparing PG^{HIGH} cells against naïve (2i/LIF) control cells. **f**, Gene-expression values projected on the UMAP plot for *Vim*, *Scd2* and *Ldhb* (upregulated in naïve 2i/LIF control cells) and *Tmsb4x*, *Mycn* and *Ldha* (upregulated in all serum/LIF samples), calculated using a two-tailed Wilcoxon rank sum test.

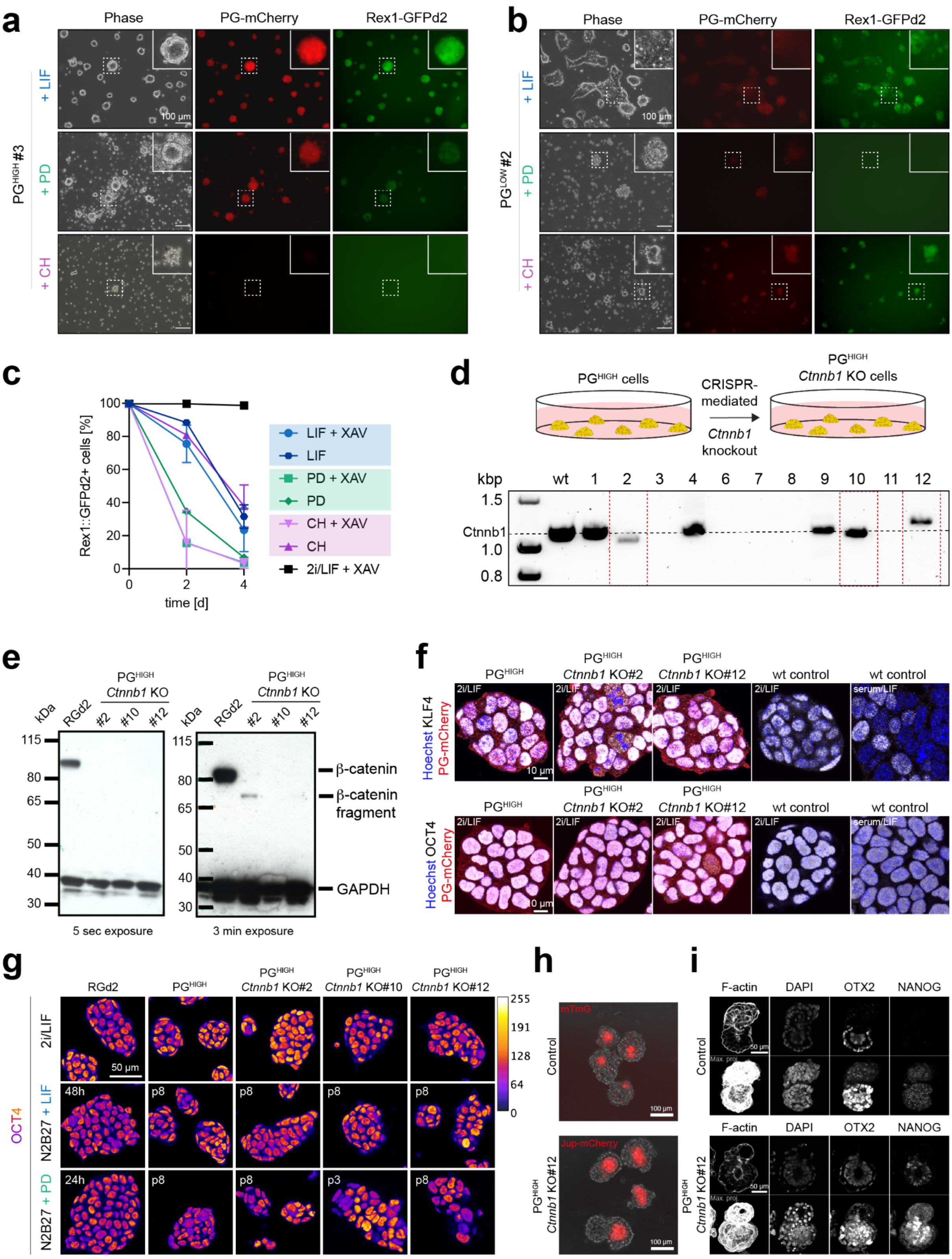

a
Phase
PG-mCherry
Rex1-GFPd2
PGHIGH #3
+ LIF
+ PD
+ CH
100 µm
b
Phase
PG-mCherry
Rex1-GFPd2
PGLOW #2
+ LIF
+ PD
+ CH
100 µm
c
Rex1::GFPd2+ cells [%]
time [d]
LIF + XAV
LIF
PD + XAV
PD
CH + XAV
CH
2i/LIF + XAV
d
PGHIGH cells
CRISPR-mediated
Ctnnb1 knockout
PGHIGH Ctnnb1 KO cells
kbp
wt 1 2 3 4 6 7 8 9 10 11 12
1.5
Ctnnb1
1.0
0.8
e
PGHIGH Ctnnb1 KO
kDa
RGd2
#2
#10
#12
β-catenin
β-catenin fragment
GAPDH
5 sec exposure
3 min exposure
f
PGHIGH
PGHIGH Ctnnb1 KO#2
PGHIGH Ctnnb1 KO#12
wt control
Hoechst KLF4 PG-mCherry
Hoechst OCT4 PG-mCherry
2i/LIF
serum/LIF
10 µm
g
RGd2
PGHIGH
PGHIGH Ctnnb1 KO#2
PGHIGH Ctnnb1 KO#10
PGHIGH Ctnnb1 KO#12
OCT4
2i/LIF
N2B27 + LIF
N2B27 + PD
48h
24h
p8
p3
50 µm
h
Control
mTmG
PGHIGH Ctnnb1 KO#12
Jup-mCherry
100 µm
i
F-actin
DAPI
OTX2
NANOG
Control
PGHIGH Ctnnb1 KO#12
Max. proj.
50 µm

Supplementary Fig. 7 | Plakoglobin sustains pluripotency independently of β-catenin.

a&b, Phase contrast and epifluorescence (PG-mCherry and Rex1-GFPd2) images of PG^{HIGH} and PG^{LOW} cells 6 days after culture in either PD, LIF or CH. Scale bar: 100 µm. Representative of 3 independent experiments. **c**, Flow cytometric analysis of the Rex1-GFPd2 reporter in microgel encapsulated cells. Cells were cultured with the sole supplementation of PD (+XAV), LIF (+XAV) or CH (+XAV). **d**, CRISPR-mediated knockout of *Ctnnb1* (β-catenin) in PG^{HIGH} cells. (see methods). Genomic knockout verification of *Ctnnb1* in PG^{HIGH} cells. *Ctnnb1* KO clone #2, #10 and #12 are highlighted by the red dotted lines. Representative of 3 independent experiments. **e**, Western blot analysis of RGd2 and PG^{HIGH} *Ctnnb1* KO (clone #2, #10 and #12) cultured under naïve conditions in 2i/LIF. β-catenin was detected in RGd2 cells but not in clone #10 and #12. Long exposure (3 minutes) revealed a small amount of truncated β-catenin in clone #2. GAPDH was used as loading control. **f**, Confocal immunofluorescence images of PG^{HIGH}, PG^{HIGH} *Ctnnb1* KO (clone #2 and #12) and wild type control cells stained for KLF4 (naïve pluripotency marker) and OCT4 (general pluripotency marker). Scale bar: 10 µm. Representative of 3 independent experiments. **g**, Representative confocal immunofluorescence images of RGd2 and PG^{HIGH} *Ctnnb1* KO (clone #2, #10 and #12) cells cultured in 2i/LIF, LIF-only and PD-only. Cells were stained for the general pluripotency marker OCT4. Scale bar: 50 µm. Representative of 3 independent experiments. **h**, Representative phase contrast images merged with epifluorescence images of mouse embryo chimeras before fixation. Control and PG^{HIGH} *Ctnnb1* KO#12 cell contribution to the embryos was confirmed by mCherry signal. Scale bard: 100 µm. **i**, Confocal immunofluorescence images of mouse embryos chimeras. 8-cell embryos were aggregated with 3-5 control (N=19 embryos) or PG^{HIGH} *Ctnnb1* KO#12 (N=24 embryos) cells cultured until a post-implantation-like state (see methods) and stained for NANOG and OTX2. Scale bar 50 µm.

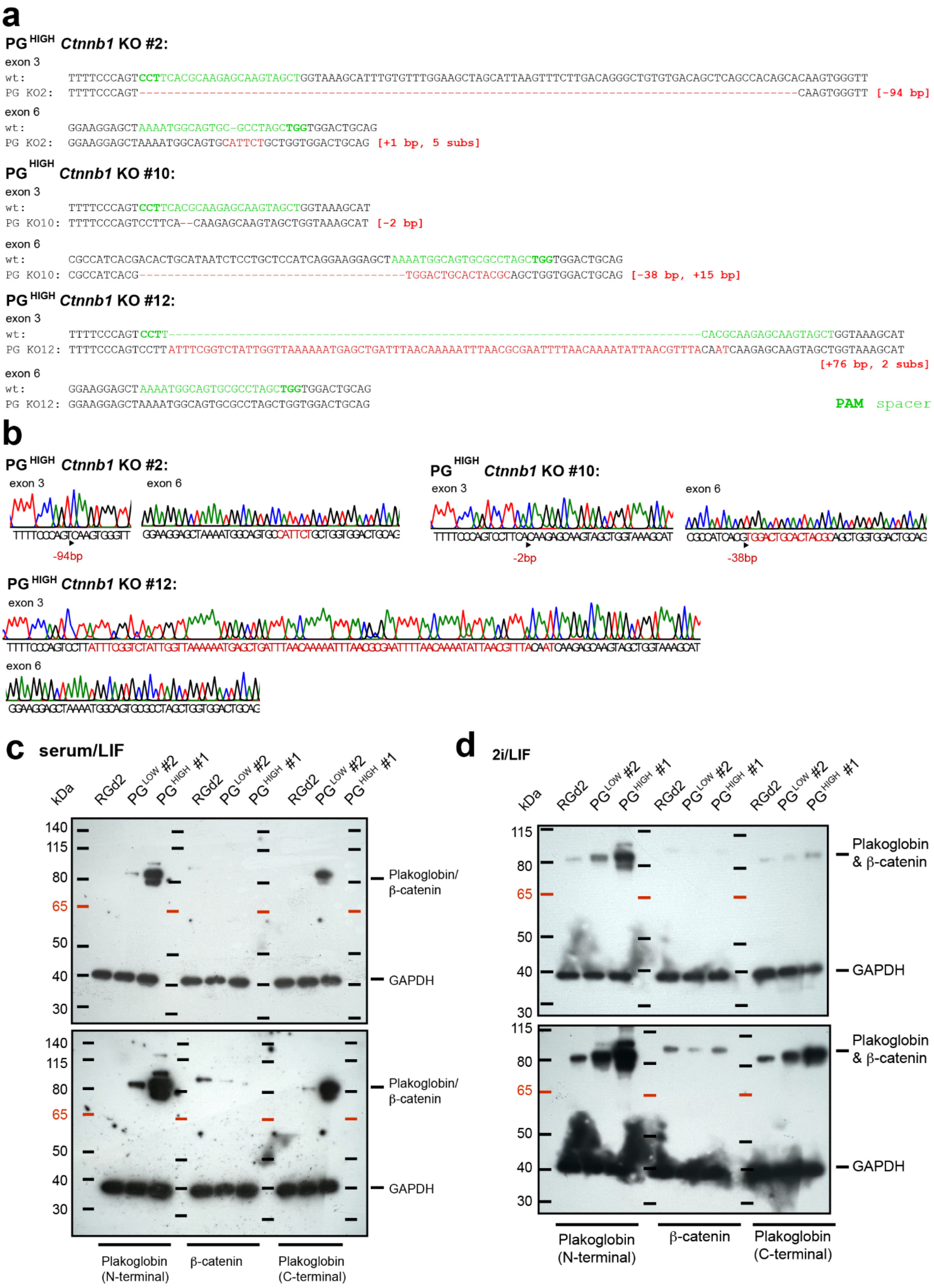
a
PG HIGH Ctnnb1 KO #2:
exon 3
wt: TTTTCCCAGTCCTTCACGCAAGAGCAAGTAGCTGGTAAAGCATTTGTGTTTGGAAGCTAGCATTAAGTTTCTTGACAGGGCTGTGTGACAGCTCAGCCACAGCACAAGTGGGTT
PG KO2: TTTTCCCAGT--CAAGTGGGTT [-94 bp]
exon 6
wt: GGAAGGAGCTAAAATGGCAGTGC-GCCTAGCTGGTGGACTGCAG
PG KO2: GGAAGGAGCTAAAATGGCAGTGCATTCTGCTGGTGGACTGCAG [+1 bp, 5 subs]
PG HIGH Ctnnb1 KO #10:
exon 3
wt: TTTTCCCAGTCCTTCACGCAAGAGCAAGTAGCTGGTAAAGCAT
PG KO10: TTTTCCCAGTCCTTCA--CAAGAGCAAGTAGCTGGTAAAGCAT [-2 bp]
exon 6
wt: CGCCATCACGACACTGCATAATCTCCTGCTCCATCAGGAAGGAGCTAAAATGGCAGTGCGCCTAGCTGGTGGACTGCAG
PG KO10: CGCCATCACG--------------------------------------TGGACTGCACTACGCAGCTGGTGGACTGCAG [-38 bp, +15 bp]
PG HIGH Ctnnb1 KO #12:
exon 3
wt: TTTTCCCAGTCCTT--CACGCAAGAGCAAGTAGCTGGTAAAGCAT
PG KO12: TTTTCCCAGTCCTTATTTCGGTCTATTGGTTAAAAAATGAGCTGATTTAACAAAAATTTAACGCGAATTTTAACAAAATATTAACGTTTACAATCAAGAGCAAGTAGCTGGTAAAGCAT
[+76 bp, 2 subs]
exon 6
wt: GGAAGGAGCTAAAATGGCAGTGCGCCTAGCTGGTGGACTGCAG
PG KO12: GGAAGGAGCTAAAATGGCAGTGCGCCTAGCTGGTGGACTGCAG
PAM spacer
b
PG HIGH Ctnnb1 KO #2:
exon 3
exon 6
-94bp
PG HIGH Ctnnb1 KO #10:
exon 3
exon 6
-2bp
-38bp
PG HIGH Ctnnb1 KO #12:
exon 3
exon 6
c serum/LIF
kDa
RGd2
PG LOW #2
PG HIGH #1
140
115
80
65
50
40
30
Plakoglobin/ β-catenin
GAPDH
Plakoglobin (N-terminal)
β-catenin
Plakoglobin (C-terminal)
d 2i/LIF
kDa
RGd2
PG LOW #2
PG HIGH #1
115
80
65
50
40
30
Plakoglobin & β-catenin
GAPDH
Plakoglobin (N-terminal)
β-catenin
Plakoglobin (C-terminal)

Supplementary Fig. 8 | Validation of *Ctnnb1* KO in PG^{HIGH} cells.

a&b, Genotyping of PG^{HIGH} *Ctnnb1* KO clones #2, #10, and #12 by Sanger sequencing with observed edits in exon 3 and 6 of *Ctnnb1* marked in red and gRNA target sequences in green (PAM in bold). **c&d** Complete membranes of Western blot analysis shown in Fig. 5e in serum/LIF (**c**) and 2i/LIF (**d)** with short (top) and long (bottom) exposure times and additional N-terminal Plakoglobin antibody.

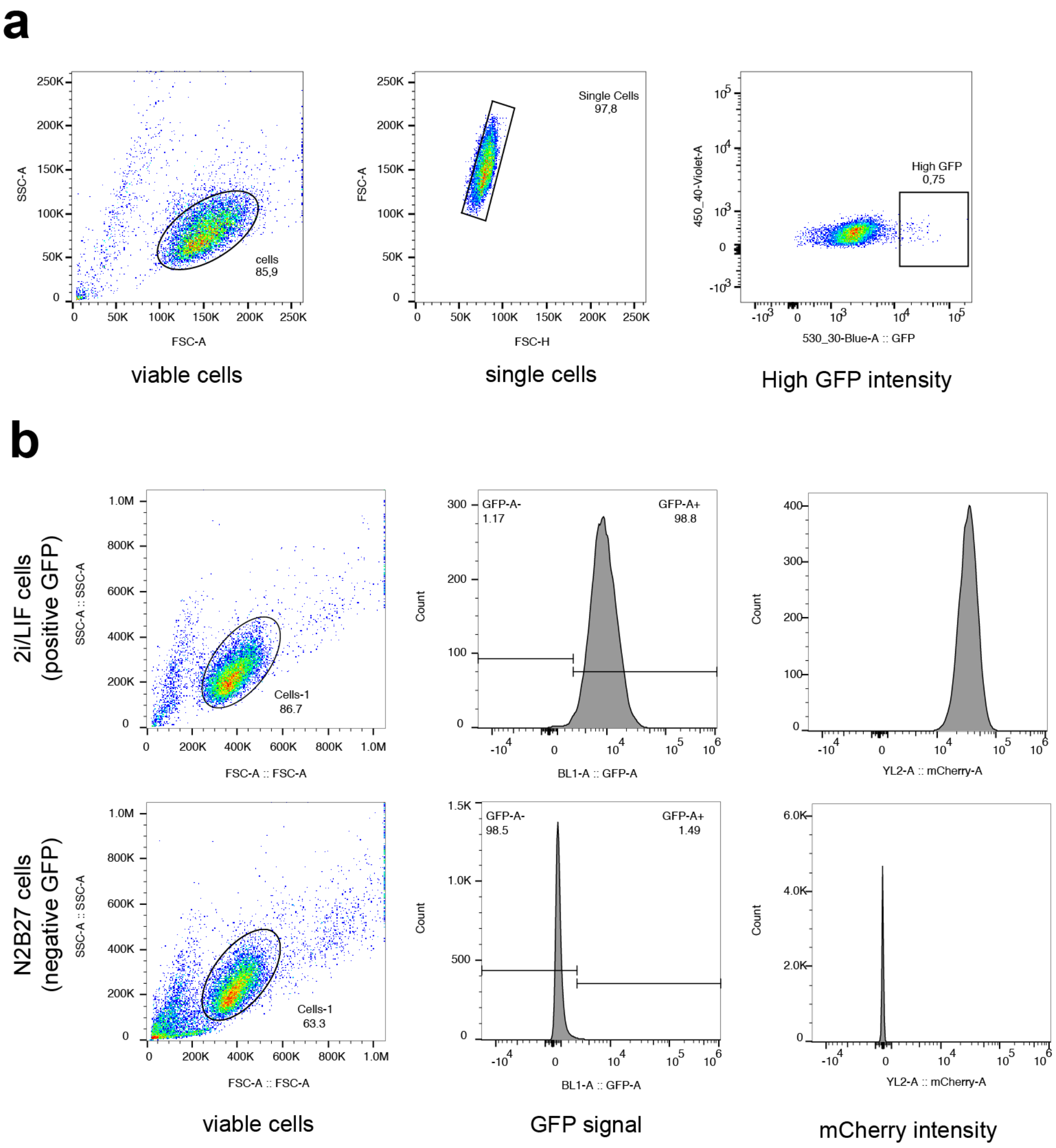

Supplementary Fig. 9 | Gating for Rex1GFPd2 cells in single cell sorting and flow cytometry analysis. **a**, Gating during single cell sorting for cell line generation. Cells were gated for cell viability, for single cells to exclude doublets and GFP intensity. **b** Gating strategy for flow cytometry analysis. Viable cells were selected by gating. The GFP signal of viable cells was plotted in histogram plots, the gate for positive and negative GFP signal was positioned in between the positive (2i/LIF) peak and the negative (N2B27) gate. This gating was applied for the analysis of the data shown in Figure 1 (e & f), 5 (f), 7 (b - e, h), S1 (f), S5 (a), S6 (a) and S7 (c).

Primary antibodies used for immunofluorescence stainings:

**Target**	**Dilution**	**Manufacturer**	**Reference**
**a-catenin**	1:200	Cell Signaling Technology	#3236S
**b-catenin**	1:200	Cell Signaling Technology	#cs8480
**E-cadherin**	1:100	Thermo Fisher Scientific	13-1900
**ESRRB**	1:300	R&D Systems,	PP-H6705-00
**KLF17**	1:500	Atlas Antibodies	HPA024629
**KLF4**	1:400	R&D Systems	AF3158
**NANOG**	1:200	Thermo Fisher Scientific	14-5761-80
**NANOG**	1:200	abcam	ab80892
**OCT4**	1:200	Santa Cruz	sc5279
**OTX2**	1:200	R&D Systems	AF1979-SP
**p120**	1:800	Cell Signaling Technology	#59854
**Plakoglobin**	1:250	abcam	ab184919
**Plakoglobin**	1:400	Cell Signaling Technology	#2309
**SOX17**	1:400	R&D systems	AF1924
**SOX2**	1:100	R&D systems	MAB2018
**T 'Brachury'**	1:200	R&D systems	AF2085
**TFCP2L1**	1:300	R&D Systems	AF5726

Primary antibodies used for western blotting:

Target	**Dilution**	**Manufacturer**	**Reference**
**b-catenin**	1:500	Cell Signaling Technology	#cs8480
**GAPDH**	1:1000	Cell Signaling Technology	#5174
**KLF4**	1:1000	R&D Systems	AF3158
**OCT4**	1:1000	Santa Cruz	sc5279
**OTX2**	1:600	R&D Systems	AF1979-SP
**Plakoglobin**	1:1000	abcam	ab184919
**Plakoglobin**	1:600	Cell Signaling Technology	#2309
**Vinculin**	1:1000	Cell Signaling Technology	#4650

Supplementary References

10. Boroviak, T. *et al.* Lineage-Specific Profiling Delineates the Emergence and Progression of Naive Pluripotency in Mammalian Embryogenesis. *Dev Cell* **35**, 366-382 (2015).

43. Mohammed, H. *et al.* Single-Cell Landscape of Transcriptional Heterogeneity and Cell Fate Decisions during Mouse Early Gastrulation. *Cell Rep* **20**, 1215-1228 (2017).

48. Stuart, H.T. *et al.* Distinct Molecular Trajectories Converge to Induce Naive Pluripotency. *Cell Stem Cell* (2019).

52. Bergmann, S. *et al.* Spatial profiling of early primate gastrulation in utero. *Nature* **609**, 136-143 (2022).
