# Supplementary material for: Plakoglobin is a mechanoresponsive regulator of naive pluripotency
Source: Nat Commun. 2023 Jul 7;14:4022. doi: 10.1038/s41467-023-39515-0 (PMC10329048; doi:10.1038/s41467-023-39515-0)
Supplement: Supplementary file 3 — Reporting Summary [file 41467_2023_39515_MOESM3_ESM.pdf]

nature portfolio

Corresponding author(s): Florian Hollfelder (fh111@cam.ac.uk) Thorsten Boroviak (teb45@cam.ac.uk)

Last updated by author(s): Mar 8, 2023

Reporting Summary

Nature Portfolio wishes to improve the reproducibility of the work that we publish. This form provides structure for consistency and transparency in reporting. For further information on Nature Portfolio policies, see our Editorial Policies and the Editorial Policy Checklist.

Statistics

For all statistical analyses, confirm that the following items are present in the figure legend, table legend, main text, or Methods section.

n/a	Confirmed	
☐	☒	The exact sample size (*n*) for each experimental group/condition, given as a discrete number and unit of measurement
☒	☐	A statement on whether measurements were taken from distinct samples or whether the same sample was measured repeatedly
☐	☒	The statistical test(s) used AND whether they are one- or two-sided<br>*Only common tests should be described solely by name; describe more complex techniques in the Methods section.*
☒	☐	A description of all covariates tested
☒	☐	A description of any assumptions or corrections, such as tests of normality and adjustment for multiple comparisons
☐	☒	A full description of the statistical parameters including central tendency (e.g. means) or other basic estimates (e.g. regression coefficient) AND variation (e.g. standard deviation) or associated estimates of uncertainty (e.g. confidence intervals)
☐	☒	For null hypothesis testing, the test statistic (e.g. *F*, *t*, *r*) with confidence intervals, effect sizes, degrees of freedom and *P* value noted<br>*Give P values as exact values whenever suitable.*
☒	☐	For Bayesian analysis, information on the choice of priors and Markov chain Monte Carlo settings
☒	☐	For hierarchical and complex designs, identification of the appropriate level for tests and full reporting of outcomes
☐	☒	Estimates of effect sizes (e.g. Cohen's *d*, Pearson's *r*), indicating how they were calculated

Our web collection on statistics for biologists contains articles on many of the points above.

Software and code

Policy information about availability of computer code

Data collection	Bulk RNA-sequencing data was collected using the Smart-seq2 protocol and datasets were obtained by sequencing on a MiSeq v2 300 cycle kit (Illumina). The read configuration for the bulk RNA sequencing was R1: 150 cycles, R2: 8 cycles, R3: 8 cycles and R4: 150 cycles. Single-cell RNA sequencing data was collected following the inDrop v3 protocol and datasets were obtained by sequencing on a Nextseq 75-cycle High Output kit (Illumina) in stand-alone mode using a 5% PhiX spike-in as an internal control. The read configuration for the bulk RNA sequencing was R1: 61 cycles, R2: 8 cycles, R3: 8 cycles and R4: 14 cycles.
Data analysis	For the bulk RNA-seq data, the fastq files were quality inspected using fastQC(v0.11.9). The STAR aligner (v2.5.2b) was used for mapping each demultiplexed paired-end file to a mm10 reference genome with a Gencode M12 gtf annotation file. Counting was achieved using featureCounts from the subread package (v1.5.0), and multi-mapped reads were discarded. Count tables were then imported in DESeq2 (v1.12.4) for differential expression analysis between the triplicate conditions and Volcano plots were obtained using the EnhancedVolcano tool (v1.0.1). For GSEA analysis, we used the stat parameter provided from the DESeq2 gene expression analysis as an input for the WebGESTALT tool (2019). For the single-cell data analysis, the BCL files were converted to fastq files using the bcl2fastq-nextseq (v0.1.0) script from Illumina. The read files were quality controlled using fastQC (v0.11.9) and de-multiplexed using Pheniqs (v2.1.0). The de-multiplexed files were used as an input for the zUMIs pipeline (v2.9.1) and mapped to a mouse GRCm38 reference genome with GRCm38.99 gtf annotation. The obtained aggregated intronic and exonic count matrices were then processed with the Scanpy tool (v1.3.7). Cells that had a low (<1%) or high fraction (>12%) of counts mapped to mitochondrial genes were excluded. Additionally, cells were filtered on the detected number of genes (between 800 and 3,000) and transcript counts (between 1,500 and 50,000). The data objects were then normalized, and transcript counts were regressed out. After scaling and principal component analysis, a dimensional reduction plot was obtained by computing a two-dimensional Uniform Manifold Approximation and Projection (UMAP) was achieved using the scanpy.tl.umap() function. Clustering was achieved using the leiden algorithm (sc.tl.leiden()). Dynamical modelling of RNA velocity was performed with the scVelo tool (v0.1.25) using the velocyto loom files obtained via the zUMIs pipeline as an input. Latent times were computed using the scVelo package.

Additionally, Fiji/ImageJ2 (v 2.9), DraftSight (v 2020), JPK Data Processing software (v 4.2), GraphPad Prism (v 9) was used to process and analyse data and Adobe Illustrator (v 2022 & 2023) was used to create figures and illustrations.

For manuscripts utilizing custom algorithms or software that are central to the research but not yet described in published literature, software must be made available to editors and reviewers. We strongly encourage code deposition in a community repository (e.g. GitHub). See the Nature Portfolio guidelines for submitting code & software for further information.

Data

Policy information about availability of data

All manuscripts must include a data availability statement. This statement should provide the following information, where applicable:

- Accession codes, unique identifiers, or web links for publicly available datasets
- A description of any restrictions on data availability
- For clinical datasets or third party data, please ensure that the statement adheres to our policy

The datasets generated during and/or analyzed during the current study are available in the GEO data repository, https://www.ncbi.nlm.nih.gov/geo/query/acc.cgi?acc=GSE197643.
Accession: GSE197643
The scripts used for RNA seq analysis can be found at https://github.com/droplet-lab/Plakoglobin/ and https://doi.org/10.5281/zenodo.7913104

Human research participants

Policy information about studies involving human research participants and Sex and Gender in Research.

Reporting on sex and gender	Human participants for this study are the couples donating embryos surplus to their requirements for assisted conception procedures. The embryos are donated with informed consent and the consent forms are anonymised before transfer from the clinic to the research premises.
Population characteristics	Sex and gender are irrelevant for this study and were not determined in the preimplantation human embryos used for this study.
Recruitment	Patients are recruited to donate embryos on a purely voluntary basis. They are provided with an information sheet summarising the project and reassurance that their choice to donate will not affect their treatment in any way and that they will receive no financial benefit either way. They are offered counselling to help them make the decision and are provided with the contact details of a senior scientist involved in a similar field of research, but with no involvement in our project.
Ethics oversight	All human embryo work for this project is covered and routinely inspected under the UK Human Fertilisation and Embryology Authority (HFEA) licence R0178 held by Jennifer Nichols.

Note that full information on the approval of the study protocol must also be provided in the manuscript.

Field-specific reporting

Please select the one below that is the best fit for your research. If you are not sure, read the appropriate sections before making your selection.

☒ Life sciences ☐ Behavioural & social sciences ☐ Ecological, evolutionary & environmental sciences

For a reference copy of the document with all sections, see nature.com/documents/nr-reporting-summary-flat.pdf

Life sciences study design

All studies must disclose on these points even when the disclosure is negative.

Sample size	No statistical method was used to predetermine sample size. Sample sizes were based on the microgel encapsulation throughput. The microfluidic encapsulations were operated at ~150 Hz (generating 150 microgels/second) with an input cell concentration of 1.6x10^6 cells/100 µl.
Data exclusions	RNA-seq data that did not pass quality control were excluded from downstream analysis (see Methods).
Replication	Experiments were performed in wild type and RGd2 cells. For Plakoglobin overexpressing cells, several clones were generated and analysed. Furthermore, experiments were repeated in the bulk sorted cells. All experiments were performed in triplicates unless otherwise stated.

Randomization	There was no allocation of test subjects for any experiments, thus randomization was not applicable to our study.
Blinding	For experimental setup and downstream analysis, the researchers needed to know culture conditions, types of cell lines, and which protocols to use. No blinding was performed. Data analyses were performed by unbiased software programs/algorithms whenever possible.

Reporting for specific materials, systems and methods

We require information from authors about some types of materials, experimental systems and methods used in many studies. Here, indicate whether each material, system or method listed is relevant to your study. If you are not sure if a list item applies to your research, read the appropriate section before selecting a response.

Materials & experimental systems

n/a	Involved in the study
☐	☒ Antibodies
☐	☒ Eukaryotic cell lines
☒	☐ Palaeontology and archaeology
☐	☒ Animals and other organisms
☒	☐ Clinical data
☒	☐ Dual use research of concern

Methods

n/a	Involved in the study
☒	☐ ChIP-seq
☐	☒ Flow cytometry
☒	☐ MRI-based neuroimaging

Antibodies

Antibodies used

Primary antibodies used for immunofluorescence staining of mouse embryonic stem cells, mouse embryos, human pluripotent stem cells, human embryos and marmoset embryos:
a-catenin – rabbit polyclonal antibody (1:200, Cell Signaling Technology, #3236S)
b-catenin – rabbit monoclonal (D10A8) antibody (1:200, Cell Signaling Technology, #cs8480)
E-cadherin – rat monoclonal (ECCD-2) antibody (1:100, Thermo Fisher Scientific, 13-1900)
ESRRB – mouse monoclonal (H6705) antibody (1:300, R&D Systems, PP-H6705-00
KLF17 – rabbit polyclonal antibody (1:500, Atlas Antibodies, HPA024629)
KLF4 – goat polyclonal antibody (1:400, R&D Systems, AF3158)
NANOG – rat monoclonal (eBioMLC-51) antibody (1:200, Thermo Fisher Scientific, 14-5761-80)
NANOG – rabbit polyclonal antibody (1:200, abcam, ab80892)
OCT4 – mouse monoclonal (C-10) antibody (1:200, Santa Cruz, sc5279)
OTX2 – goat polyclonal antibody (1:200, R&D Systems, AF1979-SP)
p120 – rabbit monoclonal (D7S2M) antibody (1:800, Cell Signaling Technology, #59854)
Plakoglobin – rabbit monoclonal (EPR17310) antibody (1:250, abcam, ab184919)
Plakoglobin – rabbit polyclonal antibody (1:400, Cell Signaling Technology, #2309)
SOX17 – goat polyclonal antibody (1:400, R&D systems, AF1924)
SOX2 – mouse monoclonal (245610) antibody (1:100, R&D systems, MAB2018)
T 'Brachury' – goat polyclonal antibody (1:200, R&D systems, AF2085)
TFCP2L1 – goat polyclonal antibody (1:300, R&D Systems, AF5726)

Primary antibodies for western blotting:
b-catenin – rabbit monoclonal (D10A8) antibody (1:500, Cell Signaling Technology, #cs8480)
GAPDH – rabbit monoclonal (D16H11) antibody (1:1000, Cell Signaling Technology, #5174)
KLF4 – goat polyclonal antibody (1:1000, R&D Systems, AF3158)
OCT4 – mouse monoclonal (C-10) antibody (1:1000, Santa Cruz, sc5279)
OTX2 – goat polyclonal antibody (1:1000, R&D Systems, AF1979-SP)
Plakoglobin – rabbit monoclonal (EPR17310) antibody (1:1000, abcam, ab184919)
Plakoglobin – rabbit polyclonal antibody (1:600, Cell Signaling Technology, #2309)
Vinculin – rabbit polyclonal antibody (1:1000, Cell Signaling Technology, #4650)

Secondary antibodies used for immunofluorescence staining and western blotting:
Donkey anti-Goat IgG (H+L) Cross-Adsorbed Secondary Antibody, Alexa Fluor™ 488 (1:1000, Thermo Fisher Scientific, A11055)
Donkey anti-Rabbit IgG (H+L) Highly Cross-Adsorbed Secondary Antibody, Alexa Fluor™ 647 (1:1000, Thermo Fisher Scientific, A31573)
Goat anti-Mouse IgG (H+L) Cross-Adsorbed Secondary Antibody, Alexa Fluor™ 555 (1:1000, Thermo Fisher Scientific, A21422)
Goat anti-Rat IgG (H+L) Cross-Adsorbed Secondary Antibody, Alexa Fluor™ 647 (1:1000, Thermo Fisher Scientific, A21247)
Goat anti-Rabbit IgG (H+L) Cross-Adsorbed Secondary Antibody, Alexa Fluor™ 555 (1:1000, Thermo Fisher Scientific, A21428)
Goat anti-Rat IgG (H+L) Cross-Adsorbed Secondary Antibody, Alexa Fluor™ 555 (1:1000, Thermo Fisher Scientific, A21434)
Rabbit anti-Goat IgG (H+L) Cross-Adsorbed Secondary Antibody, Alexa Fluor™ 647 (1:1000, Thermo Fisher Scientific, A21446)
Donkey anti-Mouse IgG (H+L) Highly Cross-Adsorbed Secondary Antibody, Alexa Fluor™ 647 (1:1000, Thermo Fisher Scientific, A31571)
Donkey anti-Rabbit IgG (H+L) Highly Cross-Adsorbed Secondary Antibody, Alexa Fluor™ 555 (1:1000, Thermo Fisher Scientific, A31572)
Donkey anti-Mouse IgG (H+L) Highly Cross-Adsorbed Secondary Antibody, Alexa Fluor™ 555 (1:1000, Thermo Fisher Scientific, A31570)

Donkey anti-Mouse IgG (H+L) Highly Cross-Adsorbed Secondary Antibody, Alexa Fluor™ 647 (1:1000, Thermo Fisher Scientific, A31571
Donkey anti-Rabbit IgG (H+L) Highly Cross-Adsorbed Secondary Antibody, Alexa Fluor™ 555 (1:1000, Thermo Fisher Scientific, A31572)
Donkey anti-Rabbit IgG (H+L) Highly Cross-Adsorbed Secondary Antibody, Alexa Fluor™ 488 (1:1000, Thermo Fisher Scientific, A21206)
HRP Goat Anti-Rabbit IgG (H+L) secondary antibody (1:10000, Cambridge Bioscience, BT204002)
Goat anti-mouse HRP secondary Antibody (1:10000, Cambridge Bioscience, AC2115)
HRP Donkey anti-Goat IgG (H+L) secondary Antibody (1:10000, Thermo Fisher Scientific, A15999)

Validation

a-catenin (Cell Signaling Technology, #3236S) – The manufacturer recommends using the antibody for WB and IF applications. The antibody has been cited in 26 publications. https://www.cellsignal.com/products/primary-antibodies/a-e-catenin-antibody/3236?site-search-type=Products&N=4294956287&Ntt=%233236s&fromPage=plp&_requestid=1489719
b-catenin – The manufacturer recommends using the antibody for WB, IF and other applications. The antibody has been cited in 1491 publications. https://www.cellsignal.com/products/primary-antibodies/b-catenin-d10a8-xp-rabbit-mab/8480
E-cadherin – The manufacturer recommends using the antibody for WB and IF applications. The antibody has been cited in 211 publications. https://www.thermofisher.com/antibody/product/E-cadherin-Antibody-clone-ECCD-2-Monoclonal/13-1900
ESRRB – The manufacturer recommends using the antibody for IHC applications, but it has also been cited for IF application in 5 publications. https://www.rndsystems.com/products/human-err-beta-nr3b2-antibody-h6705_pp-h6705-00
GAPDH – The manufacturer recommends using the antibody for WB, IF and other applications. The antibody has been cited in 5800 publications. https://www.cellsignal.com/products/primary-antibodies/gapdh-d16h11-xp-rabbit-mab/5174?_=1683132345385&Ntt=GAP&tahead=true
KLF17 – The manufacturer recommends using the antibody for WB and IHC applications. The antibody has been cited in 13 publications some of them applying the antibody for IF. https://www.atlasantibodies.com/products/antibodies/primary-antibodies/triple-a-polyclonals/klf17-antibody-hpa024629/
KLF4 – The manufacturer recommends using the antibody for WB, IF and other applications. The antibody has been cited in 46 publications. https://www.rndsystems.com/products/mouse-klf4-antibody_af3158
NANOG – The manufacturer recommends using the antibody for WB and IF applications. The antibody has been cited in 82 publications. https://www.thermofisher.com/antibody/product/Nanog-Antibody-clone-eBioMLC-51-Monoclonal/14-5761-80
NANOG – The manufacturer recommends using the antibody for WB, IF and other applications. The antibody has been cited in 293 publications. https://www.abcam.com/products/primary-antibodies/nanog-antibody-ab80892.html
OCT4 – The manufacturer recommends using the antibody for WB, IF and other applications. The antibody has been cited in 2442 publications. https://www.scbt.com/p/oct-3-4-antibody-c-10
OTX2 – The manufacturer recommends using the antibody for WB, IF and other applications. The antibody has been cited in 75 publications. https://www.rndsystems.com/products/human-otx2-antibody_af1979
p120 – The manufacturer recommends using the antibody for WB, IF and other applications. The antibody has been cited in 7 publications. https://www.cellsignal.com/products/primary-antibodies/catenin-d-1-d7s2m-xp-rabbit-mab/59854?site-search-type=Products&N=4294956287&Ntt=%2359854&fromPage=plp&_requestid=1490893
Plakoglobin – The manufacturer recommends using the antibody for WB, IF and other applications. The antibody has been cited in 8 publications. https://www.abcam.com/products/primary-antibodies/gamma-catenin-antibody-epr17310-ab184919.html
Plakoglobin – The manufacturer recommends using the antibody for WB, IF and other applications. The antibody has been cited in 53 publications. https://www.cellsignal.com/products/primary-antibodies/g-catenin-antibody/2309?site-search-type=Products&N=4294956287&Ntt=%232309&fromPage=plp&_requestid=1491767
SOX17 – The manufacturer recommends using the antibody for WB, IF and other applications. The antibody has been cited in 278 publications. https://www.rndsystems.com/products/human-sox17-antibody_af1924
SOX2 – The manufacturer recommends using the antibody for WB, IF and other applications. The antibody has been cited in 176 publications. https://www.rndsystems.com/products/human-mouse-rat-sox2-antibody-245610_mab2018
T 'Brachury' – The manufacturer recommends using the antibody for WB, IF and other applications. The antibody has been cited in 130 publications. https://www.rndsystems.com/products/human-mouse-brachyury-antibody_af2085
TFCP2L1 – The manufacturer recommends using the antibody for WB applications, but it has also been cited for IF application in 5 publications. https://www.rndsystems.com/products/human-tfcp2l1-antibody_af5726
Vinculin (Cell Signaling Technology, #4650) – The manufacturer recommends using the antibody for WB. The antibody has been cited in 223 publications. https://www.cellsignal.com/products/primary-antibodies/vinculin-antibody/4650

Eukaryotic cell lines

Policy information about cell lines and Sex and Gender in Research

Cell line source(s)

The mESC wild type cell line E14 as well as the Rex1::GFPd2 and EpiSC Oct4::GFP reporter cell lines were gifts from Prof. Austin Smith's laboratory.
Plakoglobin overexperession: The bulk cell line & clonal cell lines PG low #1-3 and PG high #1-3 were generated in this study.
PGhigh/Ctnnb1 knock-out clonal cell lines #2, #10 & #12 were generated in this study.
hPSCs: HNES1 & H9 (purchased at WiCell.com) were cultured in Prof. Jennifer Nichols laboratory.

Authentication

Pluripotency of mESCs (E14 and Rex1::GFPd2) was authenticated by immunofluorescence stainings of known pluripotency factors, on transactional level by RNA-seq and their ability to contribute to the pre-implantation blastocyst. Plakoglobin overexpression (in Rex1::GFPd2 cells) was confirmed by immunofluorescence stainings and Western blotting.
Ctnnb1 knockout (in Plakoglobin overexpressing Rex1::GFPd2 cells) was confirmed by genomic analysis, immunofluorescence stainings and Western blotting. The naive state of hPSCs (HNES1) was confirmed by KLF17 immunofluorescence staining.

Mycoplasma contamination

The cell lines testes negative for mycoplasma contamination.

Commonly misidentified lines (See ICLAC register)	No commonly misidentified cell lines were used in this study.

Animals and other research organisms

Policy information about studies involving animals; ARRIVE guidelines recommended for reporting animal research, and Sex and Gender in Research

Laboratory animals	All mice used were intercrosses of strain CD1 and obtained through natural mating. The sex of embryos and the ages of mice using mating were not concerned in this study. The mice were maintained in a state-of-the-art biofacility with daily health checks carried out by dedicated trained staff. The mice were maintained on a lighting regime of 12:12 hours light:dark with food and water supplied ad libitum
Wild animals	No wild animals were used in this study.
Reporting on sex	Sex was not considered in the study design and would not effect any of the significant results presented in this report.
Field-collected samples	No field-collected samples were used in this study.
Ethics oversight	This research has been regulated under the Animals (Scientific Procedures) Act 1986 Amendment Regulations 2012 following ethical review by the University of Cambridge Animal Welfare and Ethical Review Body (AWERB). Use of animals in this project was approved by the ethical review committee for the University of Cambridge, and relevant Home Office licences are in place.

Note that full information on the approval of the study protocol must also be provided in the manuscript.

Flow Cytometry

Plots

Confirm that:

☒ The axis labels state the marker and fluorochrome used (e.g. CD4-FITC).

☒ The axis scales are clearly visible. Include numbers along axes only for bottom left plot of group (a 'group' is an analysis of identical markers).

☒ All plots are contour plots with outliers or pseudocolor plots.

☒ A numerical value for number of cells or percentage (with statistics) is provided.

Methodology

Sample preparation	Cells were treated with Accutase to generate single cell suspensions, resuspended in either 2i/LIF medium (for sorting) or in PBS (for flow cytometry) and kept on ice until sorting/analysis.
Instrument	Cells were sorted on a 4-laser FACS Aria III (BD) with a 100 µm nozzle or on a MoFlow XDP cell sorter.<br>Flow cytometry analysis was done on a BD LSRFortessa II or Attune NxT Flow cytometer.
Software	For data collection during FACS sorts, FACSDiva version 8.3 was used.<br>For flow cytometry analysis on the BD LSRFortessa II FACSDiva and for data aquiring on the Attnune NxT the Attune NxT Software V4.2.0 was used. Data analysis was preformed with FlowJo V10.
Cell population abundance	Cells were sorted into single wells of gelatin coated 96-well plates using a strict single cell sort mask (yield mask: 0, purity mask: 32, phase mask: 16). We therefore assume that only single cells were sorted.
Gating strategy	Cells were gated based on forward and side scatter (FSC-A vs. SSC-A) and single cells were gated on FSC-A vs. FSC-H followed by SSC-A vs. SSC-H. Single highly GFP-positive cells were then sorted into 2i/LIF medium.<br>For flow cytometry analysis intact cells were gated by SCC-A vs. FCS-A and their GFP and mCherry signal was measured. Data was acquired until 10,000 viable and single cells were measured. To set gates for positive/negative GFP signal, Rex1::GFPd2 cells were cultured in either 2i/LIF (GFP positive cells) or in N2B27 for 48 hours (GFP negative cells) prior analysis and in the histogram plot a gate was placed in between these two peaks (see supplementary information).

☒ Tick this box to confirm that a figure exemplifying the gating strategy is provided in the Supplementary Information.
