# Supplementary material for: Plakoglobin is a mechanoresponsive regulator of naive pluripotency
Source: Nat Commun. 2023 Jul 7;14:4022. doi: 10.1038/s41467-023-39515-0 (PMC10329048; doi:10.1038/s41467-023-39515-0)
Supplement: Supplementary file 4 — Description of Additional Supplementary files [file 41467_2023_39515_MOESM4_ESM.docx]

File Name: Supplementary Data 1

Differential gene expression for 2Dvs3D_Bulk_diff_expression
